# A simulation study on hydrogel performance for enhanced oil recovery using phase-field method

**DOI:** 10.1038/s41598-022-06388-0

**Published:** 2022-02-11

**Authors:** Seyed Hosein Hayatolgheibi, Forough Ameli, Mohammad Reza Moghbeli

**Affiliations:** School of Chemical, Petroleum and Gas Engineering, Iran University of Science and Technology, 16846-13114 Tehran, Iran

**Keywords:** Engineering, Chemical engineering, Mathematics and computing, Computational science, Software, Statistics

## Abstract

Hydrogels are increasingly applied in oil recovery processes. This leads to more controlled flow of fluids in porous media. In this process, hydrogel is injected to the reservoir to block the high permeability areas. The trapped oil in low permeability regions, is then swept by water flooding. pH‐sensitive hydrogel microspheres were synthesized in another work of the authors, which effectively increased the oil recovery factor in experimental studies. In this communication, phase-field approach was used to simulate this process and to obtain the tuning parameters of the model including thickness of the contact surface (*є*), phase transform parameter (M_0_), and excess free energy (∧). Diffusion of hydrogels was studied by Cahn–Hilliard conservative approach and the breakage, deformation, and plugging mechanisms were analyzed, based on pressure drop variations in micromodel. Moreover, Effective parameters on oil recovery factor were analyzed. Results indicated a good agreement between experimental and modeling studies of oil recovery factor in water and hydrogel flooding with absolute errors of 2.29% and 4.06%, respectively. The recovery factor was calculated using a statistical method which was in good agreement with the modeling results. The tuned parameters of the model were reported as, *є* = 111.7 µm, M_0_ = 5 × 10^−13^ m^3^/s, $\wedge = - 0.0003$ J/m^3^.

## Introduction

During productive life cycle of a reservoir, many processes are applied to increase the oil recovery factor. The primary and secondary recovery techniques usually increase the recovery factor to 33%^1^. For viscous oils with high interfacial tension between injected fluids and oil, the recovery factor is less than this value. Researchers are work on new EOR techniques to increase sweeping and displacement efficiencies of the injected fluid^2–7^. During water injection, large channels might form which lead to defective circulation. This results in trapping a portion of oil in low permeability areas, where water injection is no longer effective and overall sweeping efficiency is reduced^1,8,9^.

In reservoirs with anisotropic permeability profile, including stratified or fractured reservoirs, secondary recovery techniques do not lead to increasing the displacement and sweeping efficiencies. In these reservoirs, injected fluid moves toward high permeability regions and leaves the low permeability areas unswept. Channeling of the injected fluid might lead to reducing the total oil recovery factor and early breakthrough of the injected fluid following that increasing the costs of separation, treatment, and water disposal. To overcome these problems, injection of gelling polymers would be a good choice^7,10–14^. In this technique, high permeability regions would plug with hydrogel and the injected fluid is diverted to low permeability zones for increasing the sweeping efficiency. More oil is produced and anomalies in the near well-bore region is blocked. As a result, injection profile would be modified and operating costs for oil production would be reduced^15^.

Various viscoelastic particles have been developed and applied for enhanced oil recovery including branched preformed particle gel (B-PPG), preformed particle gel (PPG), dispersed particle gel (DPG), and polymer elastic microsphere (PEM). PPG is synthesized using the cross linker, acrylamide monomer, and initiator, using the surface equipment. Then it is divided to small pieces, dried and sieved based on the specifications of target reservoirs^16^. After injection of swollen gels to porous media, high permeability regions are plugged and the flow is diverted to low permeability areas to increase the swept volume. This leads to increasing the displacement pressure. While this pressure is increased to extrusion pressure of the gels, particles are deformed and passed through the throats. Since they move to the depth of reservoir, the recovery factor is increased^17^. Various viscoelastic particles are represented as follows:

### Gelling polymers

Hydrogels which are used for enhanced oil recovery, are cross-linked polymers that are swollen in water without dissolving^18,19^. Some of the mostly used polymers include Polyacrylamide homopolymer (PAM) and acrylamide copolymers. Moreover, other biopolymers including guar gum, starch, chitosan, and cellulose are studied by the researchers^20^. These polymers are cross-linked with organic or inorganic substances to generate chemical hydrogels with physical bonds composed of hydrophilic interaction, van der Waals forces, and ionic complexing^21^. Hydrogels that retain anisotropic permeability of reservoirs, are swelled in water and maintain the solvent in three-dimensional structure without dissolving them. The mostly applied polymers for enhanced oil recovery include acrylamide copolymers and Polyacrylamide homopolymer (PAM). Biopolymers might also be used in this filed including cellulose, chitosan, and xanthan gum^20,21^. Effective parameters in this process include temperature, salinity, pH, and components of the gelling systems (solvent, cross linker, polymer, and reagents). Increasing the concentration of cross-linker and polymer lead to generation of a rigid hydrogel. This is due to the large number of cross links per unit of the chain length^22^. Specifications of a proper gelling system include using inexpensive environmentally friendly chemicals, good injectivity into reservoir and gelling time, and suitable biological, mechanical, and thermal stability in reservoir conditions^23^. For in situ cross-linked polymers which include gelling systems cross-linked with metal, organic compounds, and cross-linker, the polymer and cross-linking agent are simultaneously injected to the reservoir to generate hydrogel within the high permeability zones of the reservoir^24,25^. This system is successfully tested in many field applications in near wellbore and far-wellbore cases. However, difficulties for this system include properly mixing the cross-linker and polymer, gelation kinetics, plugging the whole area in the reservoir containing oil, and separation of the gelant into the reservoir^26^.

### Gelling polymers with no cross-linker

This polymer is based on a biopolymer-polysaccharide produced by fermentation. This biopolymer forms gel in absence of a cross-linker at pH values of less than 10.8. Gelation is reversible and the produced gel is dissolved while the pH value is increased.

### Pre-cross-linked polymers

This category of polymers includes microgels, performed particle gels (PPG), colloidal dispersion gels (CDG), bright water, and pH sensitive gels. Polymers are prepared at surface to be injected in the reservoir. Dispersed aggregates are injected to the reservoir and swell in the matrix in area of high permeability. Unlike in-situ cross linked polymers, they do not have the limitations of changing the gelant composition, its dilution, and reaction control problems. On the other hand, they might be filtered within the pores of the reservoir or mechanical retention might lead to high pressure drop in the system. This might lead to poor injectivity, swelling problems and plugging undesirable regions of the reservoir. Various studies have been implemented for application of pre-cross-linked polymers in the near well-bore region and deep in the reservoir rock^27^.

Gel and polymer flooding simulation studies are divided into macroscopic and microscopic categories. In macroscopic approach, there are various studies based on classical percolation theory. Yuan et al.^28^ proposed a 2D multi-component model for polymer cross-linking in the reservoir. Chen et al.^29^ performed a simulation study on oil displacement mechanism. Based on this model, Xu et al.^30^ proposed a 3D model with eight components for gel flooding. The results for saturation were improved using high-order explicit techniques. A 3D streamline method was established by Feng et al.^31^ to consider the capillary and gravity forces, and applied it for gel flooding simulation studies. Wu et al.^32^ and Cui et al.^33^ improved the proposed gel flooding models by coupling the chemical reaction and mass transfer between phases. An optimization study was performed on injection parameters for gel flooding, using starting pressure gradient method. This study was then developed for fractured reservoir by Zhao et al.^34^ using fracture-matrix coupled technique in a 3D two-phase flow. An optimization study was also performed for profile control in this reservoir. Herzig and Payatakes^35^ established a classical percolation theory. In their study, percolation coefficient was a function of percolation velocity and concentration. Particle deformation and expansion were considered by Zhang et al.^36^ in which a profile control model was proposed for fluid–solid coupling. PPG displacement was modeled by Wang et al.^37^, considering the relationship between pore throats and particles. Restarting pressure and deformation were modeled in this study. A power law model was proposed to calculate the restarting rate. Moreover, a distribution function was recommended to estimate the probability of pore plugging. Surfactant migration model was coupled with PPG flooding to describe the interaction between surfactant and PPG. One of the limitations of classical percolation theory, is that the breakthrough time is always considered one pore volume. Moreover, the particle size, deformation, and strength are not studied in this theory. Therefore, size exclusion theory was introduced to develop more accurate results. Flow modeling in pore scale is very useful to understand the transport of the fluids in the reservoir. Interfacial force could be modeled as a continuum force using diffuse interface models. In this technique, numerically resolvable layers are applied for distributing the interface discontinuities on them^38^. These methods are numerically attractive due to advantage of easier solving the Navier-Stockes equation on fixed grid in comparison to exact equation, which requires adaptive interface fitting grids. Diffuse interface models are classified into three main groups including tracking/distributed force model, continuum surface force technique ^39–41^, and phase-field based models^42–44^. Santos^45^ and Bedrikovetsky^46^ established a population equilibrium model in which particles penetrate to large pore throats or might be captured by the reservoir rocks. A modified colloidal model was proposed by You et al.^47^ in which inlet concentration was increased by the particles in accessible pores. This model led to more appropriate results for fitting the experimental data. Liu et al.^48^ upgraded the single-phase flow to two-phase flow model in which PPG migration was modeled based on the pore distribution.

Another approach for studying the displacement mechanisms in microscale, include molecular dynamics. In a recent study, snapshots of molecular dynamic simulation was applied for analyzing the whole displacement process. Laplace equation was applied to configure the oil–water interface^49^. This technique gives an insight to residual oil displacement in reservoir and provides a vision in EOR studies^50,51^. Wang^52^ applied lattice Boltzmann method for simulation of liquid flow in nanoscale porous media. Han et al.^53^ combined LBM–DEM for simulation of migration process in turbulent flow for irregular shaped particles. In this study, flow was analyzed using LBM and Smagorinsky turbulent technique. Particles interaction was characterized using DEM and particle–fluid interaction was studied using intrusive boundary conditions. Ohtsuki et al.^54^ applied a hybrid simulation model for studying the particle concentration effect on permeability. The fluid–solid interaction was studied using a coupled system. LBM was incorporated to model, for studying the fluid flow in mesoscopic level. Mechanical and geometric characteristics were analyzed using DEM at microscopic level. Interaction between solid and liquid was modeled based on boundary method. A new solver was introduced by Xiong et al.^55^ for simulation of particle–fluid interactions in large scale. Zhou et al.^56,57^ introduced a new approach for simulation of deformable PPG migration using LBM, IBM, and DEM. In this study, a functionality was developed between particle-throat diameter, elastic modulus, and critical pressure gradient. Phase-field method (PFM) is an interface capturing technique, that is widely applied due to its simplicity and accuracy for complex geometries and moving interfaces^58,59^.

In this paper, an attempt was made to use the phase-field model for simulation of hydrogel injection. This model makes it possible to track particles during hydrogel injection in micromodel. Phase-Field model together with Conn-Hilliard and Navier–Stokes flow equations makes it possible to simultaneously study on various effective parameters in flooding. These parameters include, interfacial tension, wettability, density and viscosity of various fluids, hydrogel swelling, and etc.,. Deformation of hydrogel particles and their breakage is well modeled during the movement in micro-model. Results indicated that large particles are broken at inlet of the micro-model and deformed particles with smaller diameter are moved more deeply. Also in this study, we tried to show the real conditions of the reservoir, in which the high-permeability and low-permeability areas are not considered as separated sand packs, but are continuously connected to each other and fluid transfer is possible between them. This model also allows to study the effects of temperature and gravity. Moreover, in this study swelling ratio is represented as follows:

$$ {\text{SR}} = \frac{{w_{s} - w_{d} }}{{w_{d} }} $$

where W_s_ represents the weight of the swollen hydrogel at a specified time and W_d_ denotes the weight of the dried hydrogel. The swelling ratio was obtained using experimental studies. In simulation studies, the free energy of the system was defined in such a way that increasing the volume of hydrogels due to swelling was applied in the model.

## Materials and methods

**Figure 1:**
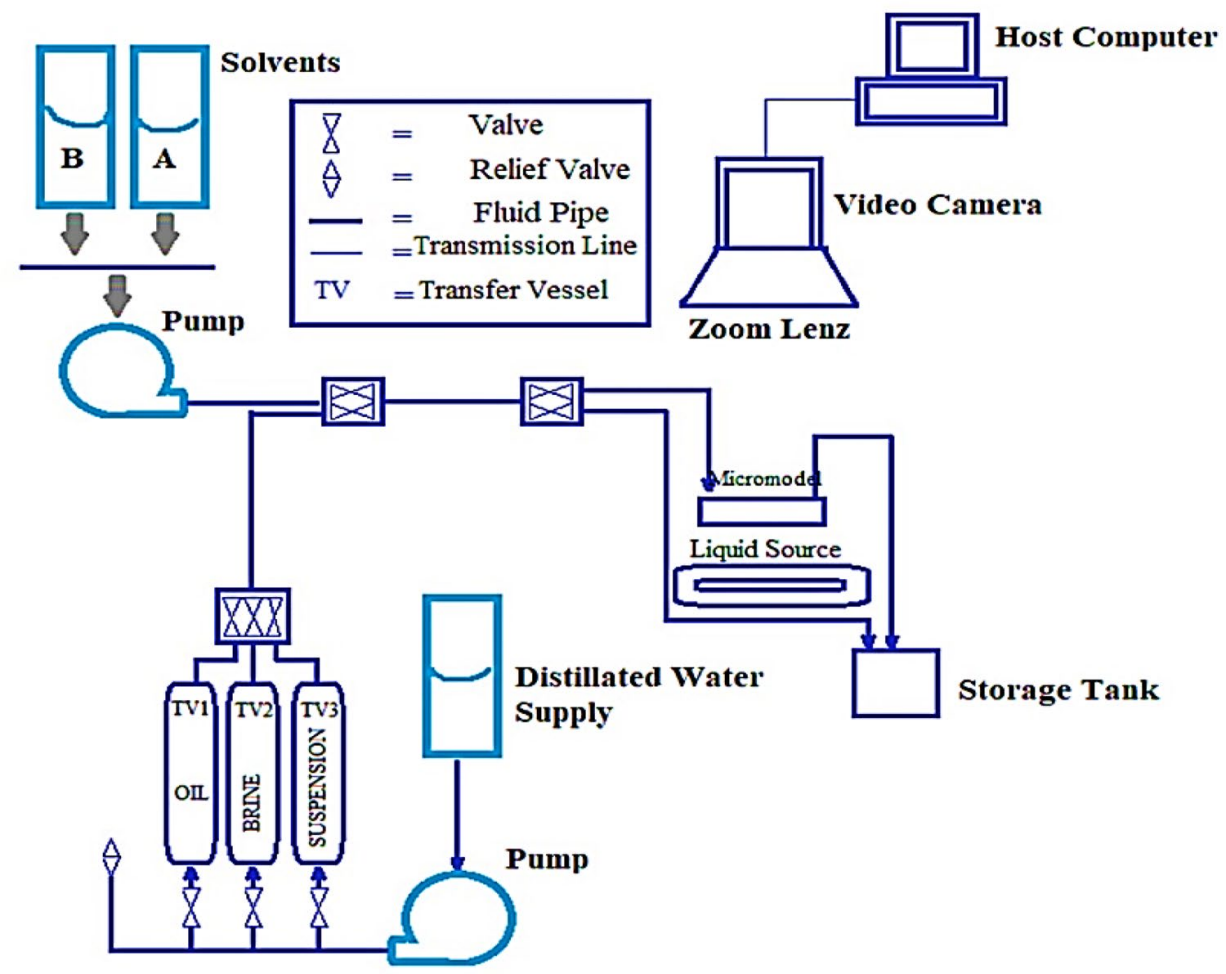
Schematic of hydrogel injection process^60^.

Experimental setup of hydrogel injection process is explained in the work of Jamali et al.^60^ and illustrated in Fig. 1. Oil samples were obtained from Siri reservoir with specific gravity of 0.85, °API gravity of 32.27, and viscosity of 16 cp. pH‐sensitive hydrogel microspheres containing silica nanoparticles were synthesized. The hydrogel solution namely, poly(acrylamide‐co‐methylenebisacrylamide‐co‐acrylic acid) was prepared with concentration of 1000 ppm and homogenized with an ultrasonic probe with power of 30 kW for 15 min. Micromodel tests were designed for injection of hydrogel with optimum formulation based on the work of Jamali et al.^60^ in which the mixer round was 443.5 rpm, AAcid/AAm wt.% was 62%, and Si nano-particle content was 0.25%.

### Micromodel experiments

A two-layer homogeneous micromodel was designed to study on increasing the oil recovery factor using the synthetized hydrogels. To do tests, water was injected to a saturated micromodel with oil. Then water was injected to micromodel. The injected water was channelized through high permeable zone. Oil recovery factor in this step was 51.46%. Hydrogel solution was then injected to micromodel. Since hydrogel particles were swelled, and high permeable region was blocked, the flow was diverted to the low permeability area, and the pressure difference between input and output was increased. For the last step, water was injected to recover the trapped oil in low permeability region. Experimental values of the recovery factor from the work of Jamali et al.^60^ are represented in Table 1.

**Table 1:** Experimental results for the oil recovery factor from the work of Jamali et al.^60^.

	Low permeable zone	High permeable zone	Total
RF (%) for 1st water flooding	0	51.46	31.84
RF (%) for hydrogel injection	1.46	69.43	39.79
RF (%) for 2nd water flooding	13.79	87.54	55.38

## Simulation of hydrogel flooding

Simulation studies was performed in two steps. Water flooding that was followed by hydrogel injection. Comsol Multiphysics software was applied for simulation of gel flooding process using phase-field approach.

### Phase-field model

Phase-field method (PFM) is a widely applied technique for complex geometries and moving interfaces, due to its simplicity and high accuracy^58,59^. Since phase-field method take use of advection techniques, they are easily implemented in three dimensions by finite element method and unstructured grids. Dissipative energies and surface tension could be easily implemented in this method. Constitutive relations including relative permeability and capillary pressure would be beneficial for flow simulation^61^. The interfaces should have four to eight cells to calculate surface tension and interface energy. There are two categories for phase-field model including conservative Cahn–Hilliard model in which diffusion is driven by the chemical potential^62^, and non-conservative Allen–Cahn model^63,64^. In phase-field model, advection term is implemented without distortion in three dimension in unstructured grids.

#### Two-phase flow equations

**Figure 2:**
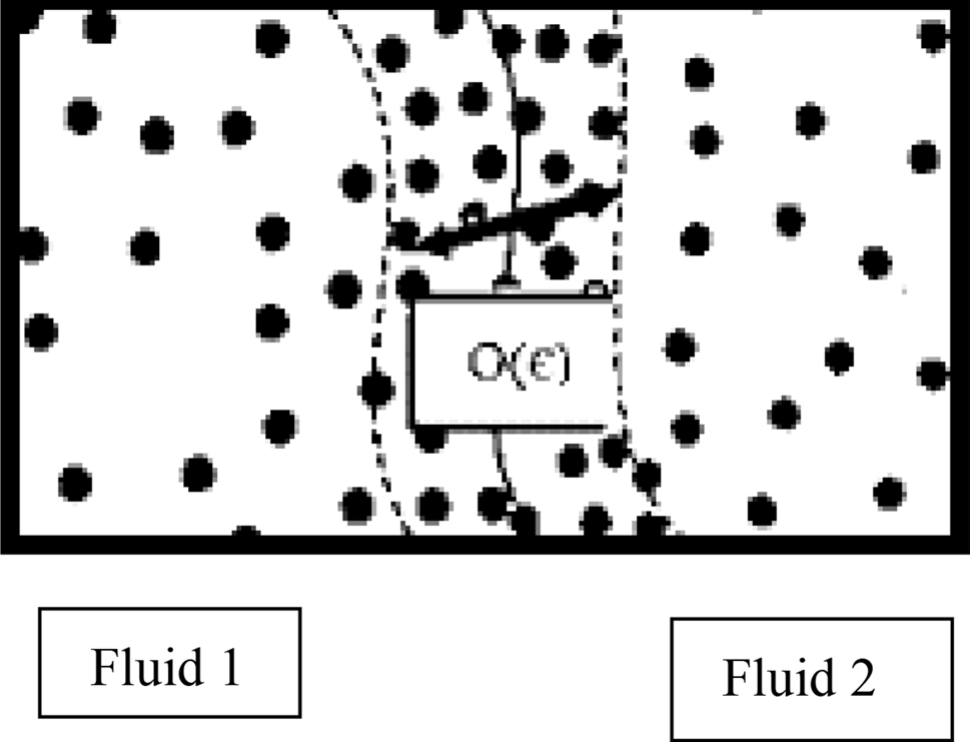
Representation of phase-field model^69^.

In this method, the two-fluid contact surface is considered as a diffusive thin layer represented in Fig. 2. In fact, the diffusive contact surface model provides a way for continuous modeling of contact surface forces, whereby the discontinuities at contact surface are propagated on a thin, numerically soluble layer. In this method, transfer equations at contact surface are replaced by continuous transfer-diffusion equations, where the emission rate is determined by the chemical potential gradient (*ϕ*) and interfacial tension is calculated based on the interfacial disturbance energy. Using these equations, contact surface displacement and deformation are calculated on a fixed network. Using the surface diffusion model for Navier–Stokes multiphase flow equations is much easier than solving them with precise equations. Cahn–Hilliard method is applied for studying conversion, formation and dissolution at contact surface^65^. In this method, the function of the free energy is minimized, which is represented in Eq. (1).

1 $$ \frac{\partial \varphi }{{\partial t}} + u \cdot \nabla \varphi = \nabla \cdot \left( {M\nabla G} \right). $$

where $\varphi$ represents the chemical potential and *u* is the fluid velocity. The mass concentration and chemical potential are related by the following Eq. ^38^:

2 $$ \varphi = \frac{{\hat{m}_{1} - \hat{m}_{2} }}{{\hat{m}_{1} + \hat{m}_{2} }} $$

Chemical potential is equivalent to the surface tension in curvature for phase-field model. *m* represents the mass concentration of the component*. G* denotes the chemical potential and is represented as follows:

3 $$ G\left( \varphi \right) = \frac{\delta F\left( \varphi \right)}{{\delta \varphi }} = \lambda \left( {\frac{{\varphi \left( {\varphi^{2} - 1} \right)}}{{\varepsilon^{2} }} - \nabla^{2} \varphi } \right) = 0 $$

Mobility is $M = M_{c} \cdot \varepsilon^{2}$, where *M*_*c*_ is the specific mobility and describes the transmission stability due to diffusion. *є* describes the thickness of the contact surface between the two fluids. λ is the mixing energy density In phase field equations, viscosity and density are calculated as follows:

4 $$ \rho = \rho_{1} v_{f1} + \rho_{2} v_{f2} $$

5 $$ \mu = \mu_{1} v_{f1} + \mu_{2} v_{f2} $$

6 $$ v_{f1} = \frac{1 - \varphi }{2},\quad v_{f2} = \frac{1 + \varphi }{2}. $$

where $\rho_{1}$ and $\rho_{2}$ are densities of fluids 1 and 2. *μ*_1_ and *μ*_2_ are the viscosity of fluids 1 and 2 and *v* is the volume fraction of each phase. Since the fluids are considered incompressible, mass and volume conservation laws are applied^62^. Average curvature (1/m) is calculated using the following equation:

7 $$ k = 2\left( {1 + \varphi } \right)\left( {1 - \varphi } \right)\frac{G}{\sigma } $$

Cahn–Hilliard equation has a curvature-dependent solution that makes it possible to use it for simulation of nucleation, evaporation, and swelling processes. Areas of high curvature at contact surface are generally high potential areas, and soluble materials move through these areas to low potential surrounding zones^38^. While the fluid transfer is involved, the chemical potential would not necessarily be uniform. Precision analysis of phase-field model is complicated, since convergency is provided by three factors including mesh size, interface surface thickness, and mobility. Mobility influences the thickness and turbulence of the boundary layers for the chemical potential. The convergence rate is determined by a dual limit, which combines the approximation of phase-field model to the real sharp surface contact physics as well as approximation of the numerical methods of phase-field model to its real solution.

#### Three-phase flow equations

In this model, the contact surface is tracked between the phases by solving the continuity and Navier–Stokes equation. The contact surface of the fluid is analyzed using chemical potential and phase-field models. The free energy is minimized to define the liquid–liquid contact surface motion. Navier–Stokes is represented as follows for the three-phase flow:

8 $$ \rho \frac{\partial u}{{\partial t}} + \rho \left( {u \cdot \nabla } \right)u = \nabla \cdot \left( { - PI + \mu \left( {\nabla u + \nabla u^{T} } \right) - \frac{2}{3}\mu \left( {\nabla \cdot u} \right)I} \right) + F_{st} + F. $$

where *P* is the pressure, $\mu$ represents viscosity, *F* shows the external force. *I* is the identity matrix. *F*_*st*_ is the surface tension calculated as follows:

9 $$ F_{st} = \mathop \sum \limits_{A,B,C} \eta_{i} \nabla \varphi_{i} $$

$\eta_{i}$ denotes the chemical potential of each phase. Boyer technique^66^ was applied to track the three phase flow contact surface.

10 $$ \frac{{\partial \varphi_{i} }}{\partial t} + \nabla \cdot \left( {\vec{u}\varphi_{i} } \right) = \nabla \cdot \left( {\frac{{M_{0} }}{{\Sigma_{i} }}\nabla \eta_{i} } \right) $$

where *M*_0_ is molecular transmittance parameter. Phase-field variables are always hold in this relation. The free energy is defined as a function of phase-field variables, based on the following relation:

11 $$ F = \sigma_{AB} \varphi_{A}^{2} \varphi_{B}^{2} + \sigma_{AC} \varphi_{A}^{2} \varphi_{C}^{2} + \sigma_{BC} \varphi_{B}^{2} \varphi_{C}^{2} + \varphi_{A} \varphi_{B} \varphi_{C} \left( {\Sigma_{A} \varphi_{A} + \Sigma_{B} \varphi_{B} + \Sigma_{C} \varphi_{C} } \right) + \wedge \varphi_{A}^{2} \varphi_{B}^{2} \varphi_{C}^{2} $$

where *σ*_*ij*_ is the surface tension between phases, *i* and *j*. $\wedge$ denotes the excess free energy of the system. $\Sigma_{i}$ is defined as follows:

12 $$ \begin{aligned} \Sigma_{A} & = \sigma_{AB} + \sigma_{AC} - \sigma_{BC} \\ \Sigma_{B} & = \sigma_{AB} + \sigma_{BC} - \sigma_{AC} \\ \Sigma_{C} & = \sigma_{BC} + \sigma_{AC} - \sigma_{AB} \\ \end{aligned} $$

### Model implementation

The properties of the two parts of micromodel are represented in Table 2. In this study, the modeling approach was finite element in Comsol Multiphysics software. For walls as impermeable surfaces with no-slip boundary condition, we have:

13 $$ u_{f} \cdot \dot{t} = u_{w} \cdot \dot{t}. $$

where $\dot{t}$ is the tangent unit vector to the surface, *u*_*f*_ is the tangential fluid velocity and *u*_*w*_ is the tangential wall velocity. Since the walls are stationary, the fluid velocity along the impermeable walls is zero ($u_{f } \dot{t} = o$).

14 $$ u_{f } \dot{n} = o $$

$\dot{n}$ is the normal vector to the surface and the fluid velocities at interface,re equal ($u_{f1} = u_{f2}$).

**Table 2:** Specifications of the under-test micromodel.

Micromodel	Throat diameter (µ)	Pore diameter (µ)	Porosity	Throat dimension (cm)
High-permeable layer	200	700	62	2 × 1
Low-permeable layer	150	480	50	2 × 1

Boundary layer mesh generation was applied in this study. This type of mesh is suitable where the fluid flow is coupled with energy and mass transfer equations. For solving the Navier–Stokes equations, the modified Newton’s method was implemented. The solution starts with the initial guess for *u*_0_.

## Result and discussion

In this study, gel flooding process was simulated using Comsol Multiphysics, based on the phase-field method to study the mechanism of hydrogel performance for increasing the oil recovery factor. The developed model was validated with experimental results. Sensitivity analysis was performed on various parameters regarding micromodel and hydrogel, to propose the functionality of the recovery factor with these variables for water flooding and gel flooding processes.

### Validation of experimental results for water flooding

**Figure 3:**
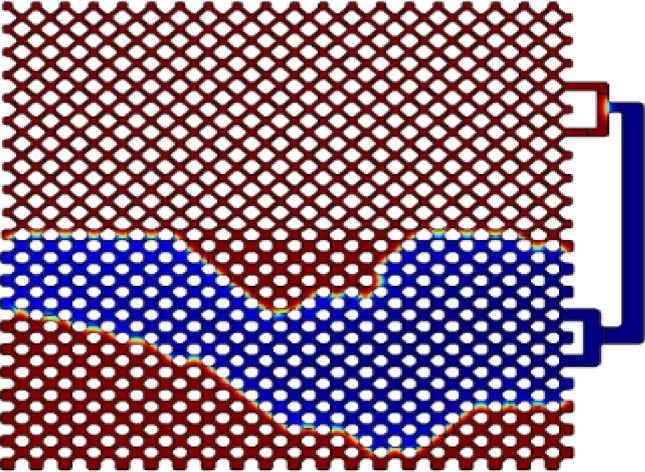
Modeling results for water flooding.

The modeling result is represented in Fig. 3 where oil and water are specified in blue and red, respectively. The results are in good agreement with experimental data from the work of Jamali et al.^60^. Comparison between recovery factor values for modeling and experimental studies are presented in Table 3. As it is obvious, in layer of low permeability, capillary force is increased and the injected fluid flows to high permeability area. Water injection is continued until the breakthrough time.

**Table 3:** Comparison of modeling and experimental results for water flooding test.

	High permeable region	Low permeable region	Total
%RF (Experimental) ^56^	51.46	0	31.84
%RF (Modeling)	52.63	0	32.57
%Error	2.29	0	2.29

### Sensitivity analysis on oil recovery factor

A statistical model was developed for prediction of oil recovery factor in water flooding process. In this section, dual effects of a number of parameters were considered.

#### Statistical model for prediction of recovery factor in water flooding

Sensitivity analysis was performed to determine the effective parameters on oil recovery factor including injection rate, wettability, and viscosities of oil and water. Using these parameters, a statistical model was proposed. The results for ANOVA analysis of variance are reported in Table 4. As it is obvious, P value is less than 0.0001, which denotes the high accuracy of this model. Coefficients of the model are reported in Table 5.

15 $$ \begin{aligned} RF & = AT + Bq + C\theta + D\mu_{o} + E\mu_{w} + FTq + GT\theta + HT\mu_{o} + JT\mu_{w} + Kq\theta + Lq\mu_{o} \\ & \quad + Mq\mu_{w} + N\theta \mu_{o} + O\theta \mu_{w} + P\mu_{o} \mu_{w} + Z \\ \end{aligned} $$

**Table 4:** ANOVA analysis of variance to determine oil recovery factor in water flooding.

	Sum of square	DOF	Mean of square errors	*F*-value	*P*-value
Model	51․95	5	10․39	1020	< 0․00001
Residual	26․47	26	1․02

**Table 5:** Coefficients of the proposed model using design expert.

Coefficient	Parameter	Coefficient	Parameter	Coefficient	Parameter
A	0.12	G	0.016	M	1.77
B	3.52	H	− 0.0015	N	6.7
C	− 10.24	J	− 0.00343	O	1.32
D	− 9.65	K	6.6	P	− 0.45
E	5.34	L	0.73	Z	37.7
F	− 0.009687				

**Figure 4:**
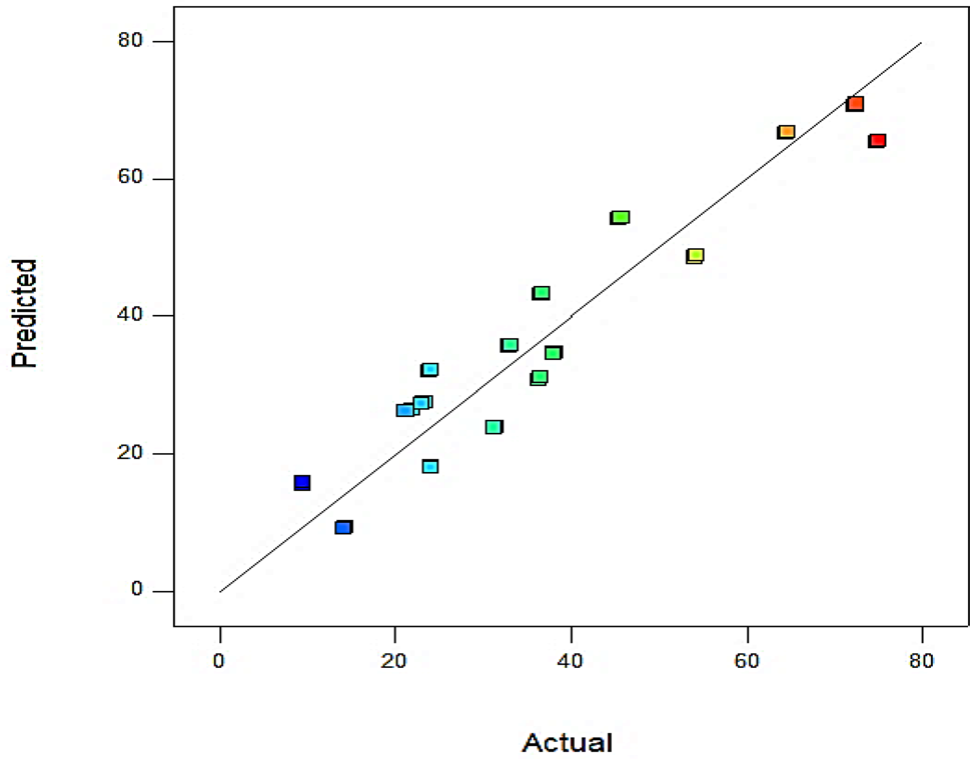
Accuracy of the developed model for predicted and actual values of RF% in water flooding.

In this equation, *q* represents the flow rate (cc/min), *Ɵ* is contact angle (degree), *µ*_*o*_ denotes oil viscosity (cp), *µ*_*w*_ is water viscosity (cp), and *T* represents temperature (°C). As it is obvious in Fig. 4, the modeling results are in good agreement with the proposed model using design expert, since most of the data are nested near the unit slop line curve.

### Capillary number

**Figure 5:**
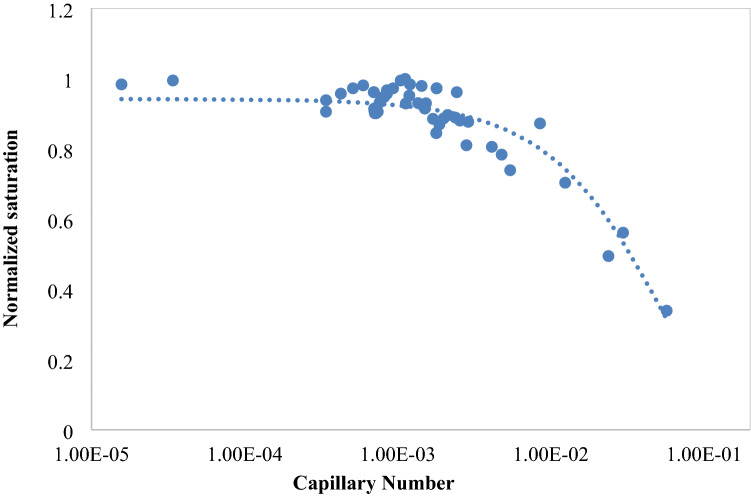
Variations of capillary number versus normalized water saturation.

Capillary number (*Ca*) defined as the ratio of viscous force to surface tension, is one of the dimensionless parameters that is used in flow interpretation. For capillary numbers more than 1, it is increased with increasing the velocity and is negligible for low Reynolds numbers. Capillary number is in the order of 10^−6^ and is about 1 in the near wellbore region and is defined as follows:

16 $$ Ca = \frac{\mu v}{{\gamma \cos \left( \theta \right)}} $$

where $\gamma$ is the surface tension. *Ca* is increased with increasing the oil recovery factor. Figure 5 illustrates the capillary number diagram in terms of normalized saturation for the modeling results in the present study.

Capillary and viscous forces are in contradiction with each other during oil recovery processes. The capillary force traps the oil in the cavities, while viscous force acts to displace oil by the injected fluid. High sweeping efficiency is an important parameter for successful EOR process which is a function of capillary number. In fact, to improve the recovery factor, capillary number is increased and the mobility ratio is reduced. According to Fig. 5, for low capillary numbers, the diagram is linear with zero slope. In critical point which is a function of wettability, permeability and pore structure, a sharp reduction is observed. Accurate representation of capillary number functionality with normalized saturation is an essential tool for simulation of oil sweeping and minimizing the residual oil saturation. Capillary number diagram in terms of normalized saturation is mostly related to reservoir heterogeneity, but it is recommended to use its model for investigation on sweeping process.

### Hydrogel flooding simulation

**Figure 6:**
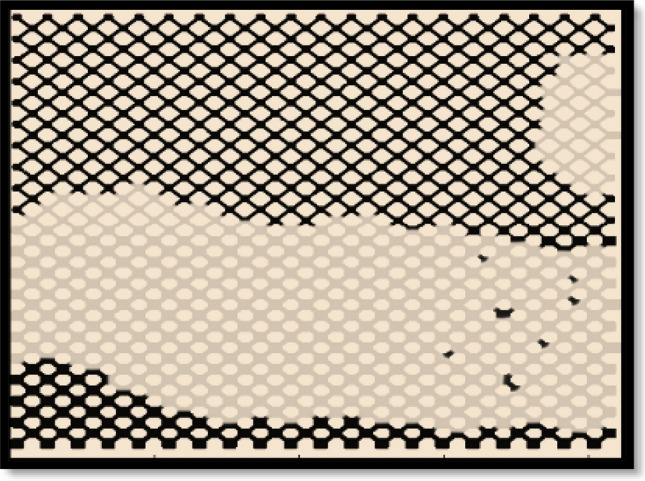
Modeling results for hydrogel injection.

As represented in previous sections, after water breakthrough, the prepared hydrogels are injected to micromodel. By injection of hydrogels, the high permeability region is blocked and water is transferred to non-swept areas of low permeability. Figure 6 illustrates the modeling result using Comsol Multiphysics software which are in good agreement with experimental study.

In simulation studies, density of hydrogels was equal to that of displacing fluid and its viscosity was 80 cp. The injection time was 1260 s in this step. The injection temperature was 25 °C, the outlet pressure was 10^5^ pa. Injection rate was 0.0005 ml/min and wettability was 40°.

**Figure 7:**
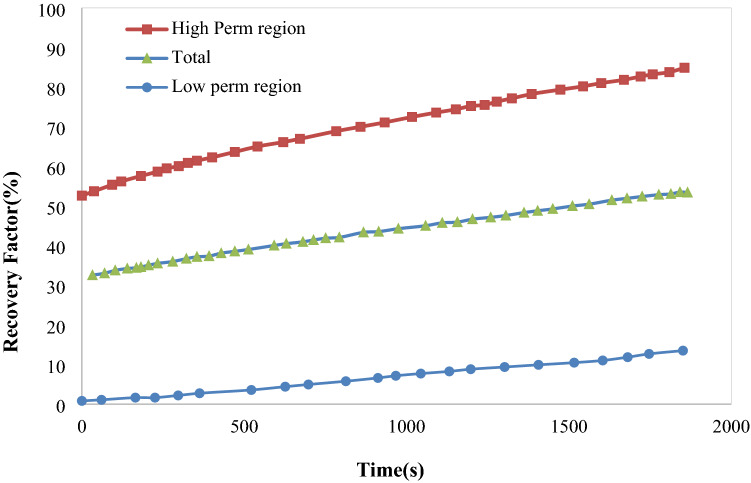
Oil recovery factor in low and high permeability regions and the whole micromodel in gel flooding.

During injection of hydrogels, they might break or transform. Table 6 represents the RF% for experimental and modeling studies. Thickness of the contact surface in simulation studies was obtained 111.7 µm. The phase transform parameter in Cahn-Hillard equation (*M*_*o*_) was equal to 5 × 10^−13^ m^3^/s, and the excess free energy ($\wedge$) was reported − 0.0003 J/m^3^. This table indicates that simulation and experimental results are in good accordance with each other. Figure 7 illustrates the variations of oil recovery factor for the whole process.

**Table 6:** Comparison of the results for experimental and simulation studies in gel flooding process.

RF	High-perm region	Low-perm region	Total
Experimental	87․54	13․79	57․05
Simulation	84․02	13․22	53․99
Error (%)	4․02	4․13	4․06

#### Mechanisms for hydrogel movement in porous media

**Figure 8:**
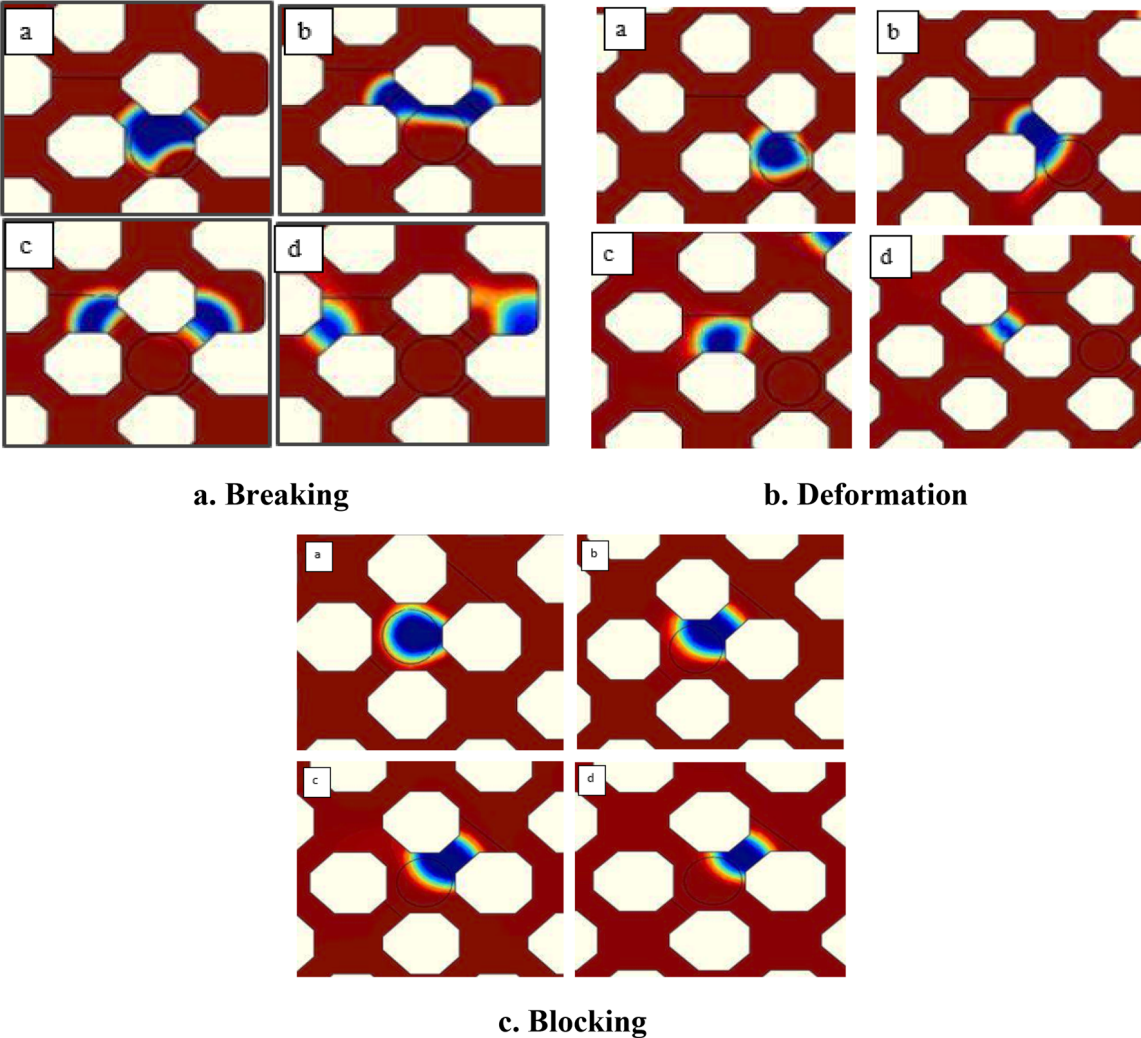
Mechanisms for hydrogel movement in porous media.

The mechanisms of hydrogel movement in porous media include breaking, deformation, and blocking, which are illustrated in Fig. 8a–c, respectively. All of these mechanisms are effective on the performance of the injected hydrogels, which are reviewed in this section.

**Figure 9:**
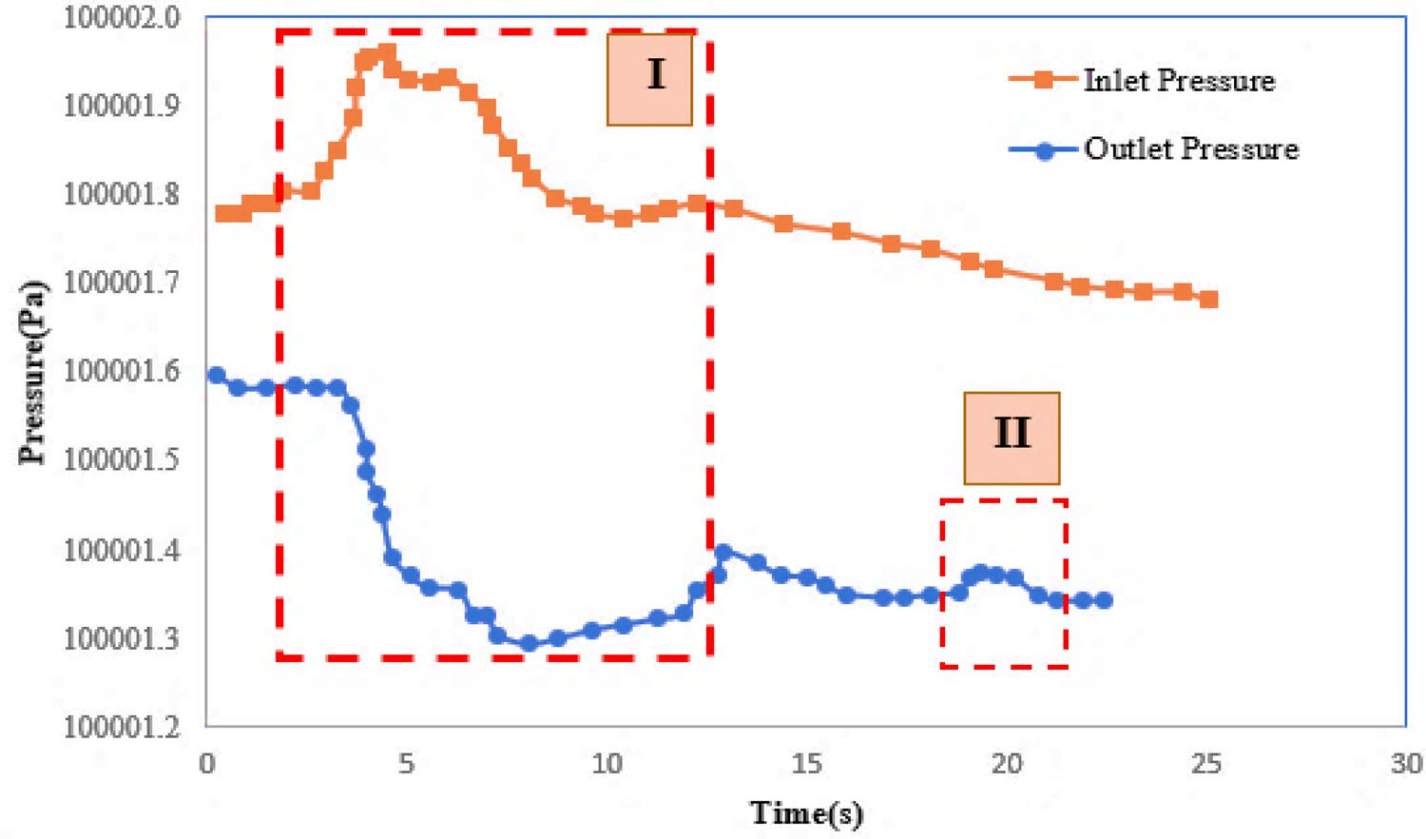
Pressure variations at inlet and outlet of the micromodel for the breakage mechanisms.

Hydrogel breakage is mostly occurred within the throats near injection point. The broken hydrogels are then penetrate more deeply in porous media. Hydrogels are broken by increasing the pressure to more than a threshold value, otherwise hydrogels would block the throats. Pressure variations at two sides of micromodel is represented in Fig. 9. Two regions are defined in this figure. In the first zone, hydrogel particle blocks the throat, temporarily. The pressure is increased at inlet and reduced at outlet. This leads to increasing the pressure difference in micromodel. As a result, hydrogel particle is deformed and broken (Fig. 8a-part b). The two generated particles are get to the next pore (Fig. 8a-part c) and one of the particles enter to the next throat (Fig. 8a-part d). As a result, the outlet pressure is slightly increased (Zone II). In all the steps for breakage and deformation, gels would plug the throat, if the pressure is not increased enough.

**Figure 10:**
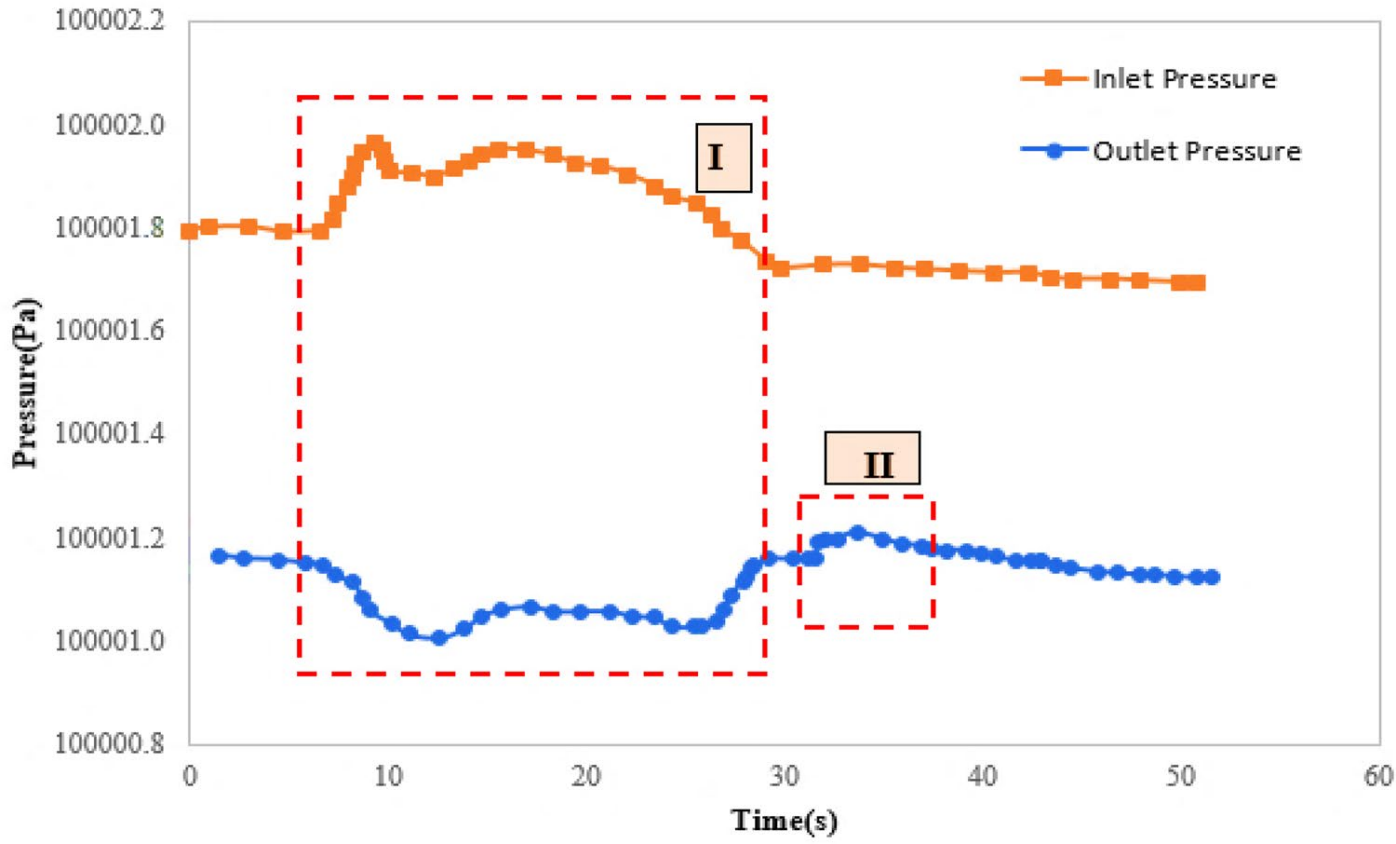
Pressure variations at inlet and outlet of the micromodel for deformation mechanism.

Hydrogels might also cross the throats by deformation, which actually happen at injection point and in medium depths of the reservoir. Figure 10 shows pressure variations at two sides of micromodel for deformation of hydrogels. Two regions are defined in this figure. In the first region, hydrogels are blocked the throat. This causes increasing the pressure difference in this region. This pressure difference leads to deformation and movement of hydrogel particles in the throat. The movement of hydrogels is continued toward the next pore and throats (Fig. 8b-part d). This is obvious with an incremental increase in outlet pressure (region II).

**Figure 11:**
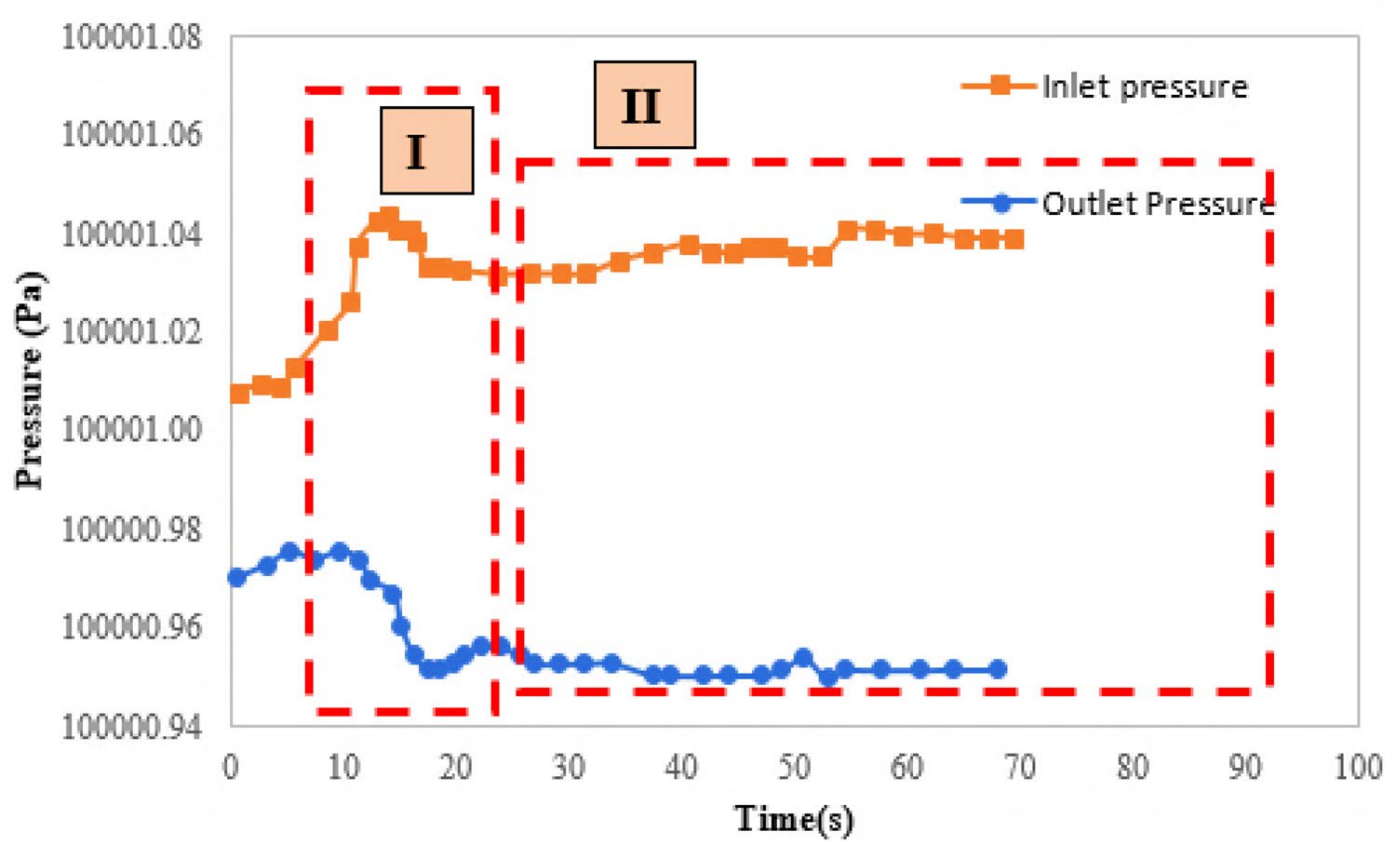
Pressure variations at inlet and outlet of the micromodel for blockage mechanism.

While the pressure variations is not enough for deformation or breakage of hydrogels, the blockage is the predominant mechanism. Blockage is mostly occurred at depth of the porous media by reducing the pressure gradient. Figure 11 shows pressure variations at two sides of micromodel for the mechanism of blockage. Hydrogel deformation is occurred at the first region by pressure variations (region I). For pressures less than the threshold value, blockage is occurred at the throat and pressure value at two sides of micromodel remains almost constant (region II). For the first region of Fig. 11, the pressure difference between two sides of micromodel is increased since hydrogels block the throat. The pressure increase leads to deformation of hydrogel, but the pressure is not high enough for breaking the hydrogels or passing them through the throats. Since the throat is blocked, the inlet and outlet pressure values, remain constant which is obvious in region II.

**Figure 12:**
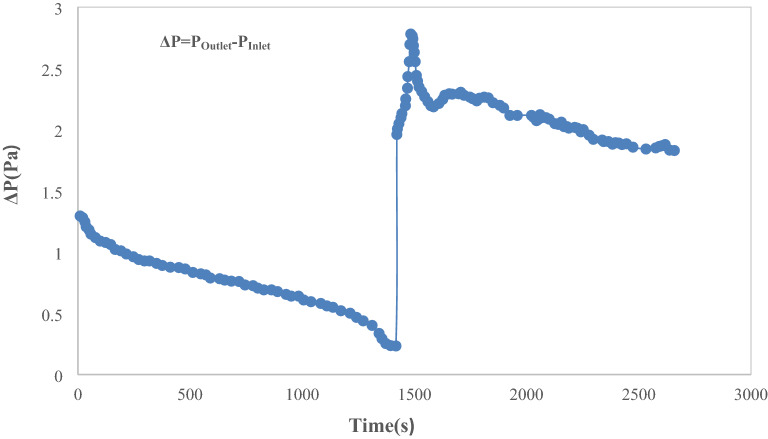
Pressure variations for the whole process of hydrogel injection.

In this section, pressure difference between the two ends of micromodel is discussed for hydrogel flooding process. The first section of Fig. 12 represents the pressure variations in micromodel for water flooding at various time steps. The pressure drop is reduced during water flooding. The next part represents the pressure variations for hydrogel flooding. After water breakthrough, during hydrogel injection, the pressure difference between inlet and outlet, represents considerable increase. Blockage of some of the throats during hydrogel injection causes this phenomenon. Moreover, there is distinctive pressure fluctuations after hydrogel injection. This is due to permanent or temporary throat blockage during hydrogels injection.

#### Pressure profile diagram

**Figure 13:**
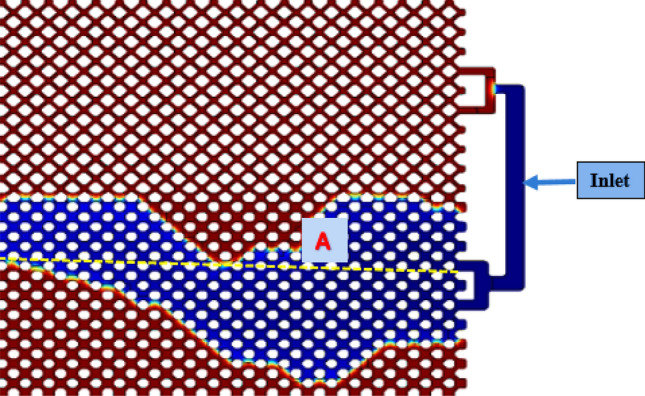
Path A to observe pressure profile during water flooding.

**Figure 14:**
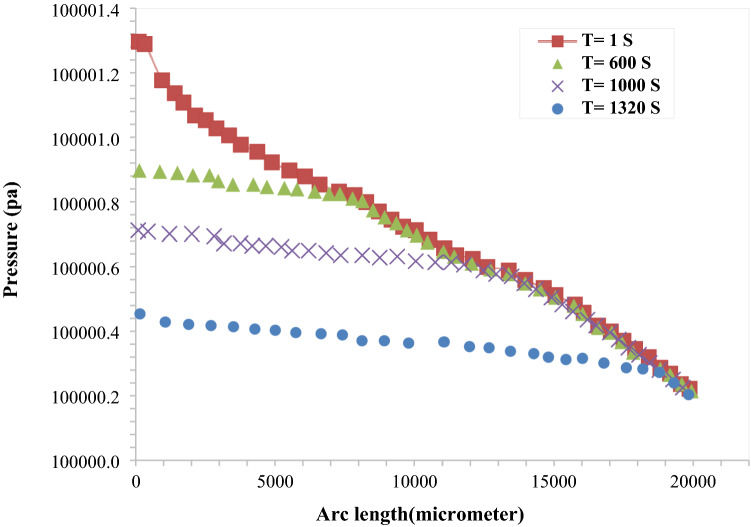
Pressure profile for water flooding at various time intervals.

To obtain pressure profile along the micromodel, the pressure variations along line A is investigated (Fig. 13). The pressure changes during water flooding is represented in Fig. 14 at various time intervals. As shown in the figure, when the initial parts of micromodel are swept, the pressure variation along the micromodel is decreased at various time steps and water is replaced by oil. At 1320 s, the pressure profile with almost monotonous slope is obtained. This means that breakthrough has occurred.

#### Sensitivity analysis on oil recovery factor for hydrogel injection

In this section, effective parameters on oil recovery factor in hydrogels injection process are analyzed. These parameters include mean diameter of hydrogel particles, hydrogel injection rate, and hydrogel viscosity.

##### Hydrogels mean radius

Hydrogel size is an important parameter in determining the penetration depth and hydrogel performance in increasing the oil recovery factor. The final radius of the injected hydrogel is a function of its initial size and the swelling ratio. Depending on the method of hydrogel synthesis and its chemical compounds, the blocking ability is different. Bai et al. ^17^stated that particles four times the throat diameter, are able to pass the throat. Larger particles should be broken before passing the throat. However, the operating conditions and hydrogel chemistry are of important parameters. Depth of penetration is a function of particle size, since it is effective on injectability of the fluid.

**Figure 15:**
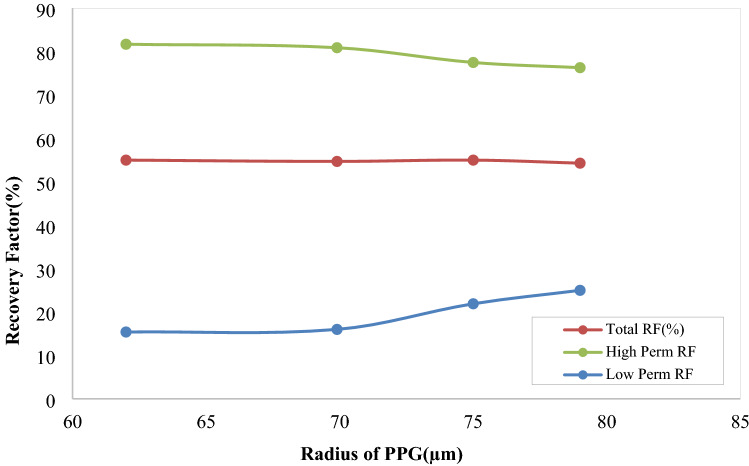
Effect of hydrogel size on oil recovery factor in high and low permeability regions and the whole micromodel.

In the present study, effect of hydrogel radius on oil recovery factor was studied. The mean radius of hydrogel particles in experimental study was 69.9 µm and the average size of throats in high and low permeability areas were 350 and 240 µm, respectively. Sensitivity analysis are performed at constant injection rate of 1e-3 cc/min and viscosity of 80cp. The results are represented in Fig. 15. Increasing the size of hydrogels led to increasing the percentage of blocked throats in high permeability region, which results in alteration the direction of the injected fluid toward the low permeability region. Therefore, the oil recovery factor would increase in this section and the amount of produced water is controlled. Of course, the size of hydrogels should not be so large that could not enter the pores. By increasing the hydrogels radii, their ability to penetrate to micromodel depth is decreased, and blockage is occurred in areas close to the injection point. This would reduce the sweeping efficiency in high-permeability region. Variations of the recovery factor in the whole micromodel, is also represented in this figure.

##### Hydrogel injection rate

**Figure 16:**
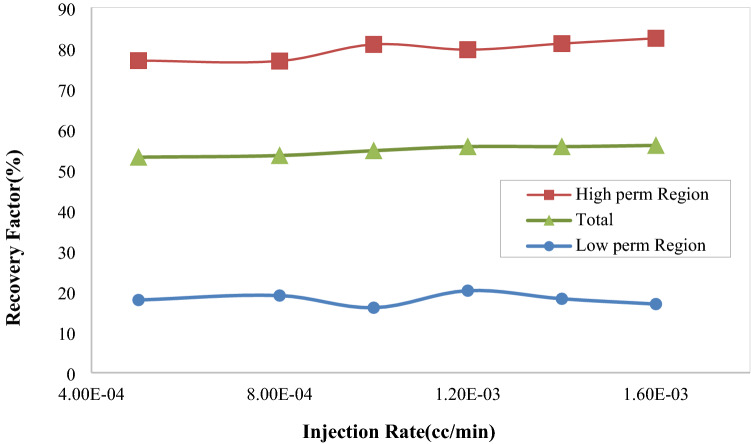
Effect of hydrogel injection rate on oil recovery factor in high and low permeability regions and the whole micromodel.

The functionality of recovery factor with injection rate is analyzed from different standpoints. The first one is the effect of injection rate on functionality of hydrogel particles, and the other is the effect of injection rate on displacement process similar to that of two-phase flow. Result of the two parameters is represented in Fig. 16. In this study, the mean radius of hydrogels was 69.9 µm and the solution viscosity was 80 cp. As represented in this figure, the recovery factor of the high permeability region is increased with increasing the injection rate up to an optimum value (1 × 10^−3^ cc/min). The recovery factor in low permeability region is at its minimum value at this point. The total oil recovery factor is increased with increasing the injection rate.

##### Hydrogel viscosity

**Figure 17:**
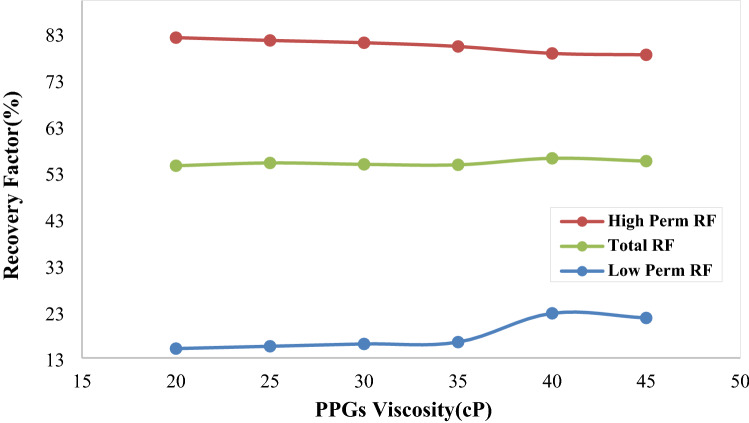
Effect of hydrogel viscosity on oil recovery factor in high and low permeability regions and the whole micromodel.

**Figure 18:**
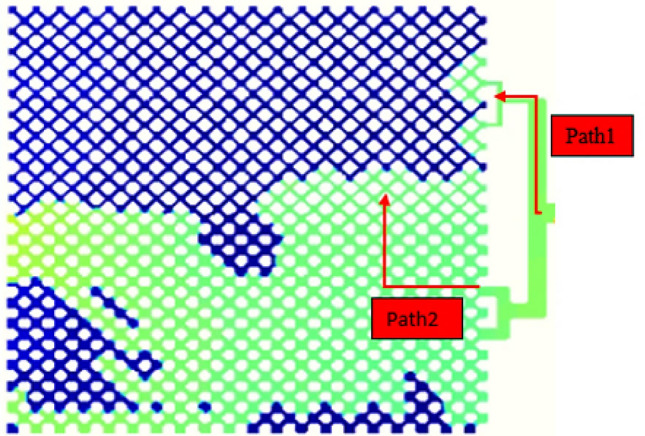
The movement mechanism of high viscosity hydrogel in micromodel.

Hydrogels are considered as a high viscosity liquid for simulation studies. To simulate solid particles, Mirzaei^67^ estimated their viscosity to be about 100 times that of a liquid. In Saghafi et al.^68^ study, the viscosity of hydrogels for simulation studies was 80 cp. The flow direction could be changed due to high viscosity of the injected hydrogels. In the present study, viscosity of the injected hydrogels was 80 cp. This value leads to an acceptable penetration depth for hydrogel particles to change the flow direction and increasing the oil recovery factor. Effect of hydrogel particles viscosity was studied on oil recovery factor in high and low permeability regions and the whole micromodel. In this study, the injection rate and hydrogel radius were 1e-3 cc/min and 69.9 µm, respectively. Effect of this parameter on oil recovery factor, is illustrated in Fig. 17. With increasing the viscosity of hydrogels, the flow in high permeability region is stopped near the injection point and leads to deviation of the flow to low permeability region. This leads to reducing the oil recovery factor in high permeability region, since hydrogels could not penetrate to the acceptable depth in micromodel. The flow resistance is increased in high permeability region and the flow is directed to low permeability part which is represented in Fig. 18 (path 1). Moreover, hydrogels are accumulated at inlet of high permeability region which steers the flow to low permeability area (path 2). With increasing the viscosity of hydrogel particles, the penetration and placement of the particles in high permeability region is reduced. This leads to reducing the oil recovery factor, since hydrogels are unable to direct the flow to low permeability area.

#### Statistical model for hydrogel injection

In this section, a statistical model is represented to determine oil recovery factor in hydrogel injection process. This model was proposed based on the effective parameters including, fluid injection rate, hydrogel size and viscosity of hydrogel solution. The results of ANOVA analysis for the high permeability region is represented in Table 7. F-values and P-values represent that the model is significant. The proposed model is represented in Eq. (17).

17 $$ \begin{aligned} RF^{ - 3} & = - 0.000133Q + 1.11783E - 09r + 1.67641E - 09\mu + 2.37196E \\ & \quad - 06Q\mu + 1.81184E - 06 \\ \end{aligned} $$

where *Q* represents the injection rate of hydrogels (cc/min), *r* denotes the hydrogel radius (*µm*), and *μ* shows the viscosity of hydrogels (*cp*). The fitting statistics are reported in Table 8.

**Table 7:** ANOVA Analysis for the recovery factor in high permeability region.

		Sum of squares	DOF	Avg. of sum of squares	*F* value	*P* value
High-perm region	Model	3.380E−14	4	8.450E−15	55.80	0.0038
	residual	1.514E−16	3	4.543E−16

**Table 8:** Fit statistics for the proposed model for high permeability region.

SD	1.231E−08	R^2^	0.9867
Mean	1.890E−06	Adjusted R^2^	0.9691
C.V. %	0.6510	Predicted R^2^	0.9057
Adeq precision	20.5770		

The same procedure was applied for the low permeability region and oil recovery factor was proposed in terms of injection rate, viscosity, and hydrogel radius by the following equation. The fitting statistics are also reported in Table 9. The results of ANOVA analysis for the low permeability region is represented in Table 10.

18 $$ RF^{ - 1.5} = 2.64543Q - 0.000010r - 0.000183\mu + 0.024399Qr + 0.020666 $$

**Table 9:** Fit statistics for the proposed model for low permeability region.

SD	0.0001	R^2^	0.9995
Mean	0.0148	Adjusted R^2^	0.9988
C.V. %	0.6081	Predicted R^2^	0.9963
Adeq precision	92.8034

**Table 10:** ANOVA Analysis for the recovery factor in low permeability region.

Source	Sum of squares	DOF	Average sum of squares	*F*-value	*P*-value
Model	0.0000	4	0.0000	1432.06	< 0.0001
Residual	2.436E-08	3	8.121E-09		

**Figure 19:**
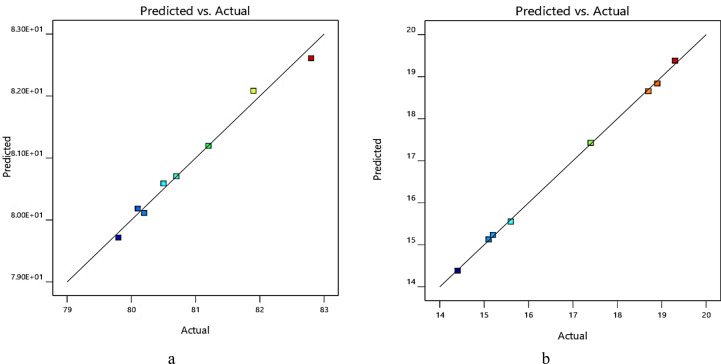
Results of the modeling and predicted values by the statistical model for (**a**) high permeability region of micromodel (**b**) low permeability region of micromodel.

Figure 19a and b, represent the results of modeling and prediction of oil recovery factor based on the proposed statistical models for high and low permeability regions, respectively. As represented in these figures, most of the data are nested near the unit slope line curve which states the high accuracy of the proposed statistical model.

## Conclusion

The present survey, focused on the performance mechanism of hydrogel for enhanced oil recovery. A simulation study was conducted for hydrogel flooding using Comsol Multiphysics software, based on the phase-filed approach. The simulation results for oil recovery factor were validated using the experimental data from another work of the authors. Sensitivity analysis was performed on oil recovery factor, based on the effective parameters in this process. Various mechanisms of hydrogel performance in the porous media were discussed and oil recovery factor was quantitatively analyzed, based on ANOVA analysis. The main results of this study are summarized as follows:

- Experimental and modeling results for water flooding and hydrogel injection processes were in good agreement with each other with absolute errors of 2.29% and 4.06%, respectively.
- In gel flooding process, the tuning parameters of the model was reported as follows:
  Thickness of the contact surface (*є*) was 111.7 µm. Phase transform parameter (M_0_) was obtained 5 × 10^−13^ m^3^/s and excess free energy ($\wedge$) was reported − 0.0003 J/m^3^.
- Increasing the hydrogel radius and viscosity has the same behavior on oil recovery factor. Hydrogel ability for in-depth penetration is reduced, and the blockage is occurred near the injection point. This would reduce the sweeping efficiency in high-permeability region and alteration the flow direction toward the low permeability region.
- The recovery factor of the high permeability region is increased with increasing the injection rate, up to an optimum value (1 × 10^−3^ cc/min). The recovery factor in low permeability region is at its minimum value at this point.
- Results of sensitivity analyses indicated the direct functionality of sweeping efficiency in low permeability region with injection rate, viscosity, and hydrogel radius. This expresses the acceptable performance of injected hydrogels for increasing the total recovery factor.

## References

[1] Han D (1995). An approach to deep development of high water-cut oil fields to improve oil recovery. Pet. Explor. Dev..

[2] Vahdanikia N (2020). Integrating new emerging technologies for enhanced oil recovery: Ultrasonic, microorganism, and emulsion. J. Petrol. Sci. Eng..

[3] Fahandezhsaadi M (2019). Laboratory evaluation of nitrogen injection for enhanced oil recovery: Effects of pressure and induced fractures. Fuel.

[4] Sofla SJD, Sharifi M, Sarapardeh AH (2016). Toward mechanistic understanding of natural surfactant flooding in enhanced oil recovery processes: The role of salinity, surfactant concentration and rock type. J. Mol. Liq..

[5] Divandari H, Hemmati-Sarapardeh A, Schaffie M, Ranjbar M (2019). Integrating synthesized citric acid-coated magnetite nanoparticles with magnetic fields for enhanced oil recovery: Experimental study and mechanistic understanding. J. Petrol. Sci. Eng..

[6] Ameli, F., Moghadam, S. & Shahmarvand, S. in *Chemical Methods* (eds Abdolhossein Hemmati-Sarapardeh *et al.*) 33–94 (Gulf Professional Publishing, 2022).

[7] Ameli F, Moghbeli MR, Alashkar A (2019). On the effect of salinity and nano-particles on polymer flooding in a heterogeneous porous media: Experimental and modeling approaches. J. Petrol. Sci. Eng..

[8] Dakuang H (2010). Discussions on concepts, countermeasures and technical routes for the redevelopment of high water-cut oilfields. Pet. Explor. Dev..

[9] Liu R, Pu W, Sheng JJ, Du D (2017). Star-like hydrophobically associative polyacrylamide for enhanced oil recovery: Comprehensive properties in harsh reservoir conditions. J. Taiwan Inst. Chem. Eng..

[10] Feng, Y. *et al.* in *International Symposium on Oilfield Chemistry.* (Society of Petroleum Engineers).

[11] Hu S, Zhang L, Yu H, Wei W, Luo J (2006). Development and prospect of the profile control/water shutoff technology in reservoir high-capacity channels. Drill. Prod. Technol..

[12] Liu Y, Bai B, Wang Y (2010). Applied technologies and prospects of conformance control treatments in China. Oil Gas Sci. Technol. Rev. d’IFP Energies Nouvelles.

[13] Ma S, Dong M, Li Z, Shirif E (2007). Evaluation of the effectiveness of chemical flooding using heterogeneous sandpack flood test. J. Petrol. Sci. Eng..

[14] Maurya NK, Kushwaha P, Mandal A (2017). Studies on interfacial and rheological properties of water soluble polymer grafted nanoparticle for application in enhanced oil recovery. J. Taiwan Inst. Chem. Eng..

[15] Tessarolli, F. G., Gomes, A. S. & Mansur, C. R. Hydrogels applied for conformance-improvement treatment of oil reservoirs. *Hydrogels, Haider S., Haider A.; Intechopen Limited: London, United Kingdom*, 69–87 (2018).

[16] Bai B (2007). Conformance control by preformed particle gel: Factors affecting its properties and applications. SPE Reserv. Eval. Eng..

[17] Bai B, Liu Y, Coste J-P, Li L (2007). Preformed particle gel for conformance control: Transport mechanism through porous media. SPE Reserv. Eval. Eng..

[18] Sengupta B, Sharma V, Udayabhanu G (2012). Gelation studies of an organically cross-linked polyacrylamide water shut-off gel system at different temperatures and pH. J. Petrol. Sci. Eng..

[19] Nishinari, K. in *Gels: Structures, properties, and functions* 87–94 (Springer, 2009).

[20] Al-Muntasheri GA (2012). Conformance control with polymer gels: What it takes to be successful. Arab. J. Sci. Eng..

[21] Zhao, H., Zhao, P., Bai, B., Xiao, L. & Liu, L. Using associated polymer gels to control conformance for high temperature and high salinity reservoirs. *J. Can. Petrol. Technol.* **45** (2006).

[22] Gehrke, S. H. in *Responsive gels: Volume transitions II* 81–144 (Springer, 1993).

[23] Vasquez, J. E. & Eoff, L. S. in *SPE Latin American and Caribbean Petroleum Engineering Conference.* (Society of Petroleum Engineers).

[24] Seright R, Lane R, Sydansk R (2003). A strategy for attacking excess water production. SPE Prod. Facil..

[25] Sydansk, R. D. & Southwell, G. in *SPE/AAPG Western Regional Meeting.* (Society of Petroleum Engineers).

[26] Seright, R. & Liang, J. in *SPE Latin America/Caribbean Petroleum Engineering Conference.* (Society of Petroleum Engineers).

[27] Choi, S. K. *A Study of a pH-Sensitive Polymer for Novel Conformance Control Applications* (2005).

[28] Shiyi Y (1991). A mathematical model of high permeability channel blockage in a heterogeneous reservoir by in-situ polymer gelation process. Shíyóu xuébào.

[29] Chen G, Zhao G, Ma Y (2004). Mathematical model of polymer linked profile control enhanced oil recovery. Qinghua Daxue Xuebao/J. Tsinghua Univ. China.

[30] Xu L, Guyenne P (2009). Numerical simulation of three-dimensional nonlinear water waves. J. Comput. Phys..

[31] Feng Q, Yuan S, Han D (2005). A new 3D streamline simulation model for weak gel driving. J. Basic Sci. Eng..

[32] Wu, X.-C. *et al.* WU Xing-Cai, PetroChina Huabei Oilfield Company, Renqiu 062552, Hebei, China; Study on nonlinearity seepage characteristic and mathematical model of movable gel. *Oil Drilling & Production Technology* **5** (2006).

[33] Cui Y, Zhu W, Sun Y, Ma Q (2009). Mathematical models of nonlinear porous flow for weak gel flooding system. J. Liaon. Tech. Univ. Nat. Sci..

[34] Wu, D. *et al.* Review of experimental and simulation studies of enhanced oil recovery using viscoelastic particles. *J. Dispers. Sci. Technol.* 1–14 (2020).

[35] Herzig J, Leclerc D, Le Goff P (1970). Flow of suspensions through porous media-new differential equation for clogged beds is derived. Ind. Eng. Chem..

[36] Zhang G., Feng Q.-H., Tong D.-K. Study on radial model of gel particle profile-control. *J. Guangxi Univ. Nat. Sci. Edn.* **3** (2009).

[37] Wang J, Liu H, Wang Z, Xu J, Yuan D (2013). Numerical simulation of preformed particle gel flooding for enhancing oil recovery. J. Petrol. Sci. Eng..

[38] Jacqmin D (1999). Calculation of two-phase Navier–Stokes flows using phase-field modeling. J. Comput. Phys..

[39] Brackbill JU, Kothe DB, Zemach C (1992). A continuum method for modeling surface tension. J. Comput. Phys..

[40] Kothe, D., Rider, W., Mosso, S., Brock, J. & Hochstein, J. in *34th Aerospace Sciences Meeting and Exhibit.* 859.

[41] Lafaurie B, Nardone C, Scardovelli R, Zaleski S, Zanetti G (1994). Modelling merging and fragmentation in multiphase flows with SURFER. J. Comput. Phys..

[42] Zaleski BNS (1996). Investigations of a two-phase fluid model. Eur. J. Mech. B/Fluids.

[43] Anderson D, McFadden G (1997). A diffuse-interface description of internal waves in a near-critical fluid. Phys. Fluids.

[44] Antanovskii LK (1995). A phase field model of capillarity. Phys. Fluids.

[45] Santos A, Bedrikovetsky P (2004). Size exclusion during particle suspension transport in porous media: Stochastic and averaged equations. Comput. Appl. Math..

[46] Bedrikovetsky P (2008). Upscaling of stochastic micro model for suspension transport in porous media. Transp. Porous Media.

[47] You Z, Badalyan A, Bedrikovetsky P (2013). Size-exclusion colloidal transport in porous media–stochastic modeling and experimental study. SPE J..

[48] Liu Y (2017). Flow of preformed particle gel through porous media: a numerical simulation study based on the size exclusion theory. Ind. Eng. Chem. Res..

[49] Luan Y, Liu B, Hao P, Zhan K, Liu J (2020). Oil displacement by supercritical CO_2_ in a water cut dead-end pore: Molecular dynamics simulation. J. Petrol. Sci. Eng..

[50] Cui M, Wang R, Lv C, Tang Y (2017). Research on microscopic oil displacement mechanism of CO_2_ EOR in extra-high water cut reservoirs. J. Petrol. Sci. Eng..

[51] Fang T (2019). Enhanced oil recovery with CO_2_/N_2_ slug in low permeability reservoir: Molecular dynamics simulation. Chem. Eng. Sci..

[52] Wang W (2021). Simulation of liquid flow transport in nanoscale porous media using lattice Boltzmann method. J. Taiwan Inst. Chem. Eng..

[53] Han K, Feng Y, Owen D (2007). Numerical simulations of irregular particle transport in turbulent flows using coupled LBM-DEM. Comput. Model. Eng. Sci..

[54] Ohtsuki S, Matsuoka T (2010). A study on the effect of particle transport on permeability in porous media by using hybrid LBM-DEM simulation. J. MMIJ.

[55] Xiong Q, Madadi-Kandjani E, Lorenzini G (2014). A LBM–DEM solver for fast discrete particle simulation of particle–fluid flows. Continuum Mech. Thermodyn..

[56] Cho Y-S, Roh SH (2018). Sol–gel synthesis of porous titania fibers by electro-spinning for water purification. J. Dispers. Sci. Technol..

[57] Zhou K (2017). An efficient LBM-DEM simulation method for suspensions of deformable preformed particle gels. Chem. Eng. Sci..

[58] Yue P, Feng JJ, Liu C, Shen J (2004). A diffuse-interface method for simulating two-phase flows of complex fluids. J. Fluid Mech..

[59] Amiri, H. A. & Hamouda, A. A. in *Proceedings of 2012 COMSOL Conference in Milan.*

[60] Jamali A, Moghbeli M, Ameli F, Roayaie E, Karambeigi M (2020). Synthesis and characterization of pH-sensitive poly (acrylamide-co-methylenebisacrylamide-co-acrylic acid) hydrogel microspheres containing silica nanoparticles: Application in enhanced oil recovery processes. J. Appl. Polym. Sci..

[61] Valvatne, P. H. & Blunt, M. J. Predictive pore‐scale modeling of two‐phase flow in mixed wet media. *Water Resour. Res.* **40** (2004).

[62] Yue P, Zhou C, Feng JJ (2007). Spontaneous shrinkage of drops and mass conservation in phase-field simulations. J. Comput. Phys..

[63] Biben T, Misbah C, Leyrat A, Verdier C (2003). An advected-field approach to the dynamics of fluid interfaces. EPL Europhys. Lett..

[64] Yang X, Feng JJ, Liu C, Shen J (2006). Numerical simulations of jet pinching-off and drop formation using an energetic variational phase-field method. J. Comput. Phys..

[65] Akhlaghi Amiri, H. A. in *COMSOL Conference 2014.*

[66] Boyer F, Lapuerta C (2006). Study of a three component Cahn–Hilliard flow model. ESAIM: Math Model. Numer. Anal..

[67] Mirzaii I, Passandideh-Fard M (2012). Modeling free surface flows in presence of an arbitrary moving object. Int. J. Multiph. Flow.

[68] Saghafi HR, Naderifar A, Gerami S, Farasat A (2016). Performance evaluation of viscosity characteristics of enhanced preformed particle gels (PPGS). Iran. J. Chem. Chem. Eng. (IJCCE).

[69] Vasilopulus, Y. *Computations of Two-phase Fluid Flows with Phase-Field Models* (2016).

